# How to compensate for frailty? The real life impact of geriatric co-management on morbi-mortality after colorectal cancer surgery in patients aged 70 years or older

**DOI:** 10.1007/s40520-024-02752-4

**Published:** 2024-08-09

**Authors:** A. Pille, H. Meillat, C. Braticevic, B. Lelong, F. Rousseau, M. Cecile, L. Tassy

**Affiliations:** 1 Service d’Oncologie médicale, Institut Paoli-Calmettes, 232 bd Sainte Marguerite, Marseille, 13009 France; 2 Service de chirurgie oncologique digestive, Institut Paoli-Calmettes, Marseille, France

**Keywords:** Comprehensive geriatric assessment, Older patients, Colorectal surgery, Enhanced recovery after surgery, Geriatric co-management

## Abstract

In Europe, CRC is the second most common cause of cancer death, and surgery remains the mainstay curative treatment. Age and frailty are associated with an increased risk of postoperative morbidity and 1-year mortality. Chronological age is not sufficient to assess the risk of postoperative complications. The CGA has been developed to better identify frail patients. Geriatric co-management have been developed to optimize the post-operative outcomes. We analyzed the real-life of geriatric co-management within an ERAS program on surgical outcomes at 90 days and oncologic outcomes at 1 year in patients aged 70 years or older after surgery for CRC. This was a retrospective study based on a prospective cohort. Fifty-one patients with a G8 score ≤ 14 were referred to geriatricians for preoperative CGA (Frail Group). They were compared with 151 patients with a G8 score ≥ 15 (Robust Group). In the Frail Group, patients were significantly older with more comorbidities than the patients in the Robust Group. Oncologic characteristics, treatments and global post-operative outcomes were comparable between the two groups. One year after surgery mortality and recurrence rates were similar between the two groups. Our study suggests that geriatric co-management is feasible and contributes to the reduction of postoperative morbimortality. Moreover, performing the CGA after G8 score screening and completion of geriatric interventions resulted in similar 90-day postoperative outcomes, in frail patients than in robust patients. Our results confirmed the benefit of geriatric co-management, involving G8 screening, CGA, and ERAS, for frail older patients undergoing surgery for CRC.

## Introduction

In Europe, CRC is the second most common cause of cancer death in both men and women [1]. Surgery is the mainstay curative treatment for CRC [2]. The Enhanced Recovery After Surgery (ERAS®) society provides up-to-date evidence-based guidelines to optimize perioperative care in various surgeries such as colorectal resection [3]. The ERAS programs were developed to improve patient care and outcomes by reducing the surgical stress response. These programs have been implemented successfully in major colorectal surgeries, resulting in reduced global morbidity and length of hospital stay (LOS) [4]. However ERAS guidelines have been validated for younger adults (< 65 years) and do not consider the age and frailty of older patients.

The incidence of cancer increases with age in particular colorectal cancer (CRC) with a peak in the incidence in the seventh and eighth decades [5]. The older population in developed countries is rapidly increasing, thereby representing a major issue in the management of CRC. Studies have shown that age alone is not an absolute contraindication for cancer surgery even in older patients [6]. Age and, more specifically, frailty are associated with an increased risk of postoperative morbidity [7] and 1-year mortality [8]. The application of ERAS guidelines for older patients has been described to be feasible and beneficial with the same results as for younger patients in terms of reducing morbidity and LOS [6, 9, 10]. 

Older patients represent a heterogeneous population with great differences in general health status and, frailty, and they often have several comorbidities. Thus, chronological age alone is not sufficient to assess the risk of postoperative complications and to optimize the perioperative management of older patients, indicating that this population requires specific perioperative cares and geriatric assessment [11]. The Comprehensive Geriatric Assessment (CGA) has been developed to better identify frail patients and assist in clinical decision-making [12]. It assesses components of a patient’s age and physiological abilities, regardless of chronological age [13], but can take several hours to complete, requires specific experience, and is not necessary for all patients [14].

Various screening methods have been developed to identify frail patients who need a full CGA before treatment. The Geriatric 8 (G8 [15]) score is one of the best screening tools for older patients with cancer who qualify for elective abdominal surgery [14]. The combination of the G8 score and CGA has been shown to be effective in predicting postoperative complications in older patients undergoing elective CRC surgery [16]. Nevertheless, the oncologic and non-oncologic surgical management of frail older patients remains challenging.

To optimize the post-operative outcomes of frail older patients, several hospitals have developed geriatric co-management. Geriatric co-management is defined as a stronger collaboration between surgical and geriatric teams with geriatrician intervention pre- and post-surgery. Geriatric co-management appears to reduce postoperative morbidity during hospitalization and at 90-days post-surgery [17–19]. 

In a previous study our group demonstrated that combining the ERAS protocol with preoperative CGA performed after G8 score screening [15] reduces the overall morbidity rate and improves the 12-month oncologic outcomes in older patients undergoing colonic cancer surgery compared with those in the control group (without ERAS protocol or CGA) [20]. Therefore, we conducted this study to investigate the real-life impact of geriatric co-manageement on patients aged 70 years or older who underwent CRC surgery.

## Materials and methods

### Patients and study design

We analyzed the real-life of geriatric co-management within an ERAS program on surgical outcomes at 90 days and oncologic outcomes at 1 year in patients aged 70 years or older after surgery for CRC. This was a retrospective study based on a prospective cohort of which geriatric characteristics were prospectively recorded.

From January 2016 to December 2019, 202 consecutive patients, aged 70 or older undergoing colonic or rectal cancer resection at the Paoli Calmettes Institute (Marseille, France), were identified from an actively maintained database. Patients who required emergency surgery or palliative surgery were not included (because of ineligibility for the ERAS protocol).

Preoperative screening according to the G8 score was systematically performed by the surgeon or coordinator nurse. Fifty-one patients with a G8 score ≤ 14 were referred to oncogeriatricians for preoperative CGA (Frail Group). They were compared with 151 patients with a G8 score ≥ 15 (Robust Group).

Informed consent was obtained from all patients before surgery, and the study was approved by the Institutional Review Board and the ethics committee. The study protocol was carried out in accordance with the 1989 World Medical Association Declaration of Helsinki.

### Patient management

A standardized protocol for enhanced recovery in accordance with ERAS recommendations was systematically applied during this period; this protocol was published previously [15, 22]. Overall 30- and 90-day morbidities were reported according to the Clavien-Dindo classification [23]. Any hospitalization of the patient within 30 days post-surgery, after being discharged home, was considered readmission. Total LOS was defined as the number of nights spent in the hospital after surgery and during any readmission within the first 30 days.

### Geriatric co-management

Geriatric co-management at Institut Paoli Calmettes is performed by oncogeriatricians who are involved in the pre- and post-operative cares of older patients, with a strong collaboration with surgeons. Referral to oncogeriatricians for preoperative care and full CGA assessment was based on the G8 score performed by surgery team. In post-operative care, referral to oncogeriatricians is based on the clinical judgment by the surgical team and need, especially to manage comorbidities, pain, and medication.

### G8 score

The G8 score [15] includes eight items: nutritional and mobility assessment, drug consumption, neuropsychological problems, and self-perception of health.

### Comprehensive geriatric assessment and geriatric interventions

Table 1 summarizes the CGA items and scales and geriatric interventions.

**Table 1:** CGA and geriatric interventions

Domain	Assessment	Cut-off value	Intervention
Social situation			Referral to social worker Social support Rehabilitation center
Multimorbity	Medical record review Clinical examination *Charlson score*		
Medication (polypharmacy)	Medication list	≥ 5	Medication review (discontinuation, change in dosage, or type of medication)
Functional status	Activities of Daily Living scale (ADL [18]) Instrumental Activities of Daily Living scale (IADL [19])	Loss ≥ 1	Visiting nurse Social support Rehabilitation center
Mobility assessment and fall risk	Fall in the last year Balance on one leg Timed Up&Go test [24]	Fall ≥ 2 Balance on one leg < 5s Timed up and Go test > 20s	Referral to physiotherapist
Cognitive function and delirium risk	Mini Mental State Examination (MMSE [21]) Mini-Cog [22] Delirium history	MMSE < 27/30 Mini-COG scoring algorithm	Peroperative caregiver Confusion Assessment Method (CAM)^27^
Emotional status	4 item Geriatric Depression Scale (mini GDS^23^)	mini-GDS score ≤ 1/4	Psychological care
Nutritional status	Mini Nutritional Assessment Short Form (MNA-SF [20]) Measurement of current weight Weight loss (whether voluntary or not) compared to normal weight Body Mass Index (BMI)	MNA-SF score < 17/30 Recent weight loss > 10% BMI < 21 kg/m2	Referral to dietician Nutritional supplement
Sarcopenia probability	Grip-test	Male < 26 kg Female < 16 kg	Referral to physiotherapist

*ADL: activities of daily living, BMI: body mass index, CAM: Confusion Assessment Method, GDS: Geriatric Depression Scale, IADL: instrumental activities of daily living, MMSE: Mini Mental State Examination, MNA-SF: Mini Nutritional Assessment Short Form*

Delirium prevention included a free accompanying person bed during the entire period of hospitalization, even in intensive care units. The surgical health care team was trained to perform the Confusion Assessment Method (CAM) [27] to detect post-operative delirium. The CAM was performed twice a day during hospitalization. In the case of malnutrition or probable sarcopenia, oral nutritional supplement was prescribed directly during the oncogeriatric consultations. Post-operative rehabilitation was arranged during oncogeriatric consultations depending on oncogeriatrician opinion of the CGA data (isolation, sarcopenia, functional status, etc.). Completion of geriatric interventions was assessed during whole patients cares.

An additional oncogeriatric consultation was planned for 1 month after surgery and/or in the case of a theoretical indication for adjuvant therapy.

### ERAS protocol and surgical treatment

Our institutional ERAS protocol was published previously [21]. 

### Oncologic treatment

The stage of colon cancer was defined according to the seventh edition of the pathological TNM classification [24], and adjuvant chemotherapy was discussed at weekly team meetings. The indications for medical oncological adjuvant treatment in our institution were constant for the entire study period. Patients with stage III or IV disease were systematically advised to receive adjuvant chemotherapy and were analyzed in our study. In the absence of a clear consensus for patients with stage II disease, they were excluded from the analyses for oncologic treatment. Chemotherapy agents included oral fluorouracil (5FU); intravenous fluorouracil/leucovorin (5FU/LV); leucovorin, fluorouracil, and oxaliplatin (FOLFOX); and leucovorin, fluorouracil, and irinotecan (FOLFIRI). First-line cetuximab or bevacizumab was only used for patients with metastatic cancer. The schedule of adjuvant chemotherapy for each patient was decided by the oncologist and the surgeon in accordance with the oncogeriatrician and the patient and considering the patient recovery status.

### Postoperative surveillance

Both groups were followed up for at least 1 year or until death with a follow-up outpatient evaluation at 7 10 days after discharge and then every 3 months for the first year. Adverse event questioning, physical examinations, computed tomography scans, and serum carcinoembryonic antigen and blood tests were performed at each follow-up visit. All deaths that occurred in the first year after surgery were considered, and causes were classified as either CRC-specific or non-cancer-related mortality.

### Statistical analysis

All statistical analyses were performed considering a significance level (α) of 0.05 using R software (R foundation for Statistical Computing, Vienna, Austria). Data were summarized by means and standard deviations, medians and ranges, or counts and frequencies as appropriate. The characteristics of both groups were compared using Chi-squared test (with Yates correction if the number was < 10) or exact Fisher test (if the number was < 5) for qualitative variables and Student’s test for quantitative variables.

## Results

### Patient characteristics

A total of 202 patients were included in the analysis: the Robust Group (G8 score ≥ 15) included 151 patients and the Frail Group (G8 score ≤ 14 and CGA) included 51 patients. Unsurprisingly, according to the G8 score screening, the patients in the Frail Group were significantly older with more frequent malnutrition and, preoperative anemia and more comorbidities than the patients in the Robust Group (Table 2).

**Table 2:** Preoperative patients characteristics

	Robust Group *N* = 151			Frail Group *N* = 51		*p* value
	N	%		N	%	
*Gender*						
Female	58		38.4	24	47	0.399
Male	93		61.6	27	53	
Age (years)*	73		(70–89)	82	(70–94)	**< 0.001**
G8 score*	15		(15–17)	12	(1–14)	**< 0.001**
Charlson score*	2		(2–12)	3	(2–11)	0.249
Comorbidities	56		37.1	37	72.6	**< 0.001**
Cardiovascular	32		21.2	22	43.1	**0.001**
Respiratory	19		12.6	18	35.3	**0.001**
Diabetes	16		10.6	11	21.6	0.063
BMI (kg/m2)*	25.4		(17.2–40.6)	26.6	(15.6–36.1)	0.680
Malnutrition	17		11.3	15	29.4	**0.004**
Preoperative anemia	34		22.5	37	72.6	**< 0.001**

BMI: body mass index, *expressed as median (range)

### Geriatric assessment

In the Frail Group, a CGA was systematically performed before surgery. Geriatric characteristics are summarized in Table 3. In this group, the median age was 82 years, and the oldest patient was 94 years old. Overall, the patients in the Frail Group presented malnutrition (54.9%), and probable sarcopenia (47.1%). Only eleven patients (20.8%) had an abnormal Activities of Daily Living (ADL) index (ADL ≤ 5). However, 35 patients (66%) had an abnormal instrumental functional status (Instrumental ADL ≤ 7). Polypharmacy was found in 36 patients (70.6%) in relation to comorbidities, and a medications review found that medication prescription optimization could be achieved for more than half of them.

At the end of the CGA, geriatric interventions were recommended by the oncogeriatricians to correct frailty. Between 60% and 100% of these interventions were completed (Table 3). Concerning psychological care, only 60% completed the recommendations, with patient refusal the most common reason for not completing the interventions. Seven patients (30.4%) did not consult a dietician before surgery owing to a lack of time. Completion was highest when measures were prescribed directly during oncogeriatric consultations (i.e. visiting nurse, oral nutritional supplement, rehabilitation center, and medications review). Although 40 patients (77.8%) were defined as being at risk for confusion and 37 patients (72.5%) were defined as being at risk for fall, no delirium syndrome or fall was observed during hospitalization.

**Table 3:** Preoperative CGA

Frail Group patients geriatric characteristics				
	Frail Group *N* = 51			
	N		%	
ADL*	6		(0–6)	
IADL*	5.5		(1–8)	
Autonomy loss	9		17.6	
Isolation	27		52.9	
Polypharmacy	36		70.6	
Malnutrition	28		54.9	
MNA*	9		(3–14)	
Depression	20		39.2	
mini-GDS*	0		(0–4)	
Delirium risk	40		78.4	
mini-COG*	4		(0–5)	
MMSE*	26		(8–29)	
Sarcopenia probability (Grip test Male < 26 kg Female < 16 kg)	24		47.1	
Male grip test (kg)*	24.5		(8–42)	
Female grip test (kg)*	15		(8–26)	
Fall risk	37		72.5	
**Intervention and support**				
	Recommended		Completed	
	N	%	N	%
Referral to social worker	27	52.9	25	92.6
Social support	20	39.2	15	75
Postoperative caregiver	39	76.5	32	82.1
Rehabilitation center	25	49	20	80
Medications review	51	100	51	100
Referral to dietician	23	45.1	16	69,6
Oral nutritional supplement	33	64.7	33	100
Enteral nutrition	3	5.9	3	100
Psychological care	15	29.4	9	60
Delirium prevention	43	84.3	39	90.7
CAM	51	100	51	100
Referral to physiotherapist	31	60.8	26	83.9
Visiting nurse	48	94.1	46	95.8

ADL: activities of daily living, BMI: body mass index, CAM: Confusion Assessment Method, GDS: Geriatric Depression Scale, IADL: instrumental activities of daily living, MMSE: Mini Mental State Examination, MNA-SF: Mini Nutritional Assessment Short Form, *expressed as median (range)

### Oncologic characteristics

Oncologic characteristics and treatments were comparable between the two groups (Table 4).

**Table 4:** Oncologic characteristics and treatments

	Robust Group *N* = 151		Frail Group *N* = 51		*p* value
	N	%	N	%	
*Localisation*					
Rectum	52	34.4	16	31.4	0.55
Colon	99	65.6	35	68.6	
*Neo-adjuvant treatment*					
Preoperative chemo-radiotherapy	24	15.9	12	23.5	0.28
Preoperative chemotherapy	12	8	4	7.8	0.78
*Surgical technique*					
Robotic	43	28.5	9	18	0,11
Laparoscopy	108	71.5	42	82	
*Stade (post-operative)*					
Stade I-II	97	64.2	36	70.6	0.44
Stade III-IV	54	35.8	15	29.4	
Including metastasis	14	9.2	3	5.9	0.79
*Adjuvant treatment*					
Postoperative chemotherapy indication	45	29.8	15	29.4	0.98
Postoperative chemotherapy	40	88.8	9	60	0.02

### Postoperative morbidity

Global post-operative morbidity rate at 90 days was similar in both groups, and post-operative complications were not more severe in the Frail Group despite these patients being older with more comorbidities. The median total LOS was slightly longer in the Frail Group (7 days versus 6 days; *p* = 0.004), despite Frail Group patients being discharged more frequently to rehabilitation centers (Table 5).

**Table 5:** Surgical and oncologic outcomes

Surgical outcomes (global postoperative morbidity at 90 days)					
	Robust Group *N* = 151		Frail Group *N* = 51		*p* value
	N	%	N	%	
Total LOS*	6	(2–37)	7	(3–26)	0.004
*Discharge disposition*					
Home	138	92	35	66	< 0.001
Rehabilitation center	12	8	18	34	
*Post-operative complications J90*					
Global post-operative complications	37	24.7	18	34	0.095
Clavien dindo 1–2	28	18.7	14	26.4	0.11
Clavien dindo 3–4	9	6	4	7.5	0.75
Reoperation	4	2.7	3	5.7	0.38
Readmission	7	4.7	4	7.5	0.48
Non-scheduled consultation	4	2.7	2	3.8	0.65
***Oncologic outcomes (1 year)***					
Recurrence 1 year	11	7.3	1	1.9	0.19
Mortality 1 year	0	0	2	3.8	0.07
Follow-up lost 1 year	2	1.3	1	1.9	1

### Oncologic outcomes

One year after surgery mortality and recurrence rates (distant metastasis or loco-regional recurrence) were similar between the two groups. Two patients in the Frail Group died during the first year after surgery: one owing to metastatic breast carcinoma and one following to a second colic localization. Both patients died 11 months after surgery. Eleven patients (7.3%) in the Robust Group had a recurrence within1 year, ten had distant metastasis and one had loco-regional recurrence. In the Frail Group, only one case of distant metastasis was observed. No non-cancer-related death was observed (Table 5).

## Discussion

Our study suggests that geriatric co-management is feasible and contributes to the reduction of postoperative morbimortality after CRC surgery within an ERAS program for frail older patients. Moreover, performing the CGA after G8 score screening and completion of geriatric interventions resulted in similar 90-day postoperative morbidity in frail older patients than in robust older patients. Our results are consistent with previous studies. CGA and geriatricians’ involvement in the perioperative care of older patients who underwent oncologic surgical treatment are becoming increasingly important to reduce post-operative morbi-mortality [25, 26]. This is a retrospective study based on a prospective cohort in which geriatric characteristics are prospectively recorded. This study was based on a monocentric cohort which allowed efficient co-management with full involvement of oncogeriatricians from the early stage of medical care and the surgical health care team. Considering that patients were consecutively included and that there were no exclusion criteria, this study can be considered as a real-life study.

Frailty increases post-operative morbi-mortality. This increased post-operative mortality in older patients has mainly been attributed to differences in early mortality [27]. Early mortality might be mainly due to postoperative complications [8, 28]. Previous studies revealed that older patients with CRC who survive the first year after surgery may have the same overall cancer-related survival rates as younger patients. Correctly treating older patients is becoming increasingly challenging in an aging population, and geriatric co-management appears to be specifically interesting for elective surgery. The CGA allows the detection of frailty markers, which enables the implementation of appropriate measures to address each marker. This co-management benefits both frail and robust patients.

Geriatric co-management appears to improve postoperative outcomes in patients undergoing oncologic and non-oncologic surgery. Its benefits have been widely demonstrated in orthopedic surgery [19, 29] and, more recently, in vascular surgery [18, 30], oncologic surgery [31, 32], and in trauma centers [30]. This was the first study to report the 90-day surgical outcomes and 1-year oncologic outcomes following geriatric co-management. Our results revealed that pre-operative CGA after G8 score screening achieved similar outcomes for frail older patients than for robust older patients. These results are consistent with recent studies on geriatric co-management [31]. In our study, the global post-operative morbidity in frail older patients was not only similar to that in robust older patients but similar, if not lower, than that in younger patients in the literature [10]. 

A short-term consequence of reducing postoperative morbidity is shortening of the LOS. In our study, the LOS was only one day longer in the Frail Group than in the Robust Group (7 days vs. 6 days), which is consistent with the literature [9, 33]. The LOS for older patients in the literature is still longer than that for younger patients (4–12 vs. 3.9–12 days) [10]. 

Frail Group patients were more often discharged to rehabilitation centers than robust older patients (34% vs. 8%). Although we expected the LOS of Frail Group patients to be longer, LOS was not correlated with the discharge mode. Considering discharge to rehabilitation centers only, the LOS for the Frail Group was shorter than that for the Robust Group (median 9 vs. 11 days). Compared with home discharge, the median LOS for the Robust Group was 5 days, whereas it was 7 days for the Frail Group. Rehabilitation was often indicated for the Frail Group patients during CGA, suggesting that performing CGA results in a reduced LOS for frail older patients anticipating rehabilitation. When rehabilitation was not recommended, home discharge was nevertheless optimized through nurse visits or social support and systematic telephonic follow-up at days 1, 7 and 30 after discharge.

To our knowledge, this is the first study to report the completion rates of geriatric interventions after CGA. We obtained very high completion rates; more than 80% of most of the recommended interventions were completed. We did not observe delirium syndrome or falls in the Frail Group despite the high risk of delirium and fall (85.1% and 72.5%, respectively). Moreover, delirium or falls were not observed in the Robust Group neither suggesting that the interventions benefited all patient; therefor, training of health care personnel and participation in perioperative protocols reduce frequent complications such as falls and delirium.

Additionally, CGA and support did not extend the median time from surgical consultation to surgery, as shown in a previous study [20]. These results demonstrate that CGA and, more importantly, geriatric intervention completion are feasible before surgery. However, selection bias was present in our cohort as patient undergoing emergency and palliative surgeries were not included; however, this was a real-life consecutive cohort without exclusion criteria.

This is a retrospective study based on a prospective cohort in which geriatric characteristics are prospectively recorded. This study was based on a monocentric cohort which allowed efficient co-management with full involvement of oncogeriatricians from the early stage of medical care and the surgical health care team. Considering that patients were consecutively included and that there were no exclusion criteria, this study can be considered as a real-life study.

A limitation of this study is that the methodology did not allow for the assessment of the specific impact of CGA independently of the ERAS protocol. Another limitation is that the study was not designed as a randomized study. However, frailty has been identified as a post-operative complication risk increasing morbi-mortality [7, 14, 34]. Moreover, CGA [35, 36], the G8 score [15], and the ERAS protocol [3] have been individually validated, and in this study, we evaluated the benefit of combining these tools. The strength of this study it was a real-world study that revealed that the completion rate of geriatric interventions is high globally.

An economic evaluation of this protocol could be interesting. On the one hand, geriatric assessment and corrective and preventative measures are time-consuming and represent additional costs. On the other hand, reducing LOS and avoiding post-operative complications represent a potential cost reduction. The costs associated with the geriatric perioperative unit have been evaluated for orthopedic surgery, and the results revealed a positive income statement regardless of emergency or standard procedures, even when paramedical staff was increased [37]. The ERAS protocol appears cost-effective in the medium term as well. A study have shown that implementing an ERAS program is expensive; however, costs are offset by reduced postoperative resource utilization with an overall cost saving [38]. 

When it comes to invasive treatment, older patients are more concerned about preserving their quality of life than their life expectancy. Consequently, a study to assess the quality of life and patient satisfaction following geriatric co-management might be of interest.

In conclusion, our results confirmed the benefit of geriatric co-management, involving G8 screening, CGA, and ERAS, for frail older patients undergoing surgery for CRC. Geriatric co-management appears to be specifically interesting for elective surgery. A prospective multicentric study is needed to validate our findings. Moreover, a randomized trial could be valuable; however, depriving frail patients of the ERAS protocol and geriatric care is likely to be unethical.
